# An Insertion Mutation in *Bra032169* Encoding a Histone Methyltransferase Is Responsible for Early Bolting in Chinese Cabbage (*Brassica rapa* L. ssp. *pekinensis*)

**DOI:** 10.3389/fpls.2020.00547

**Published:** 2020-05-12

**Authors:** Shengnan Huang, Li Hou, Wei Fu, Zhiyong Liu, Chengyu Li, Xiang Li, Hui Feng

**Affiliations:** Department of Horticulture, Shenyang Agricultural University, Shenyang, China

**Keywords:** Chinese cabbage, bolting, insertion mutation, histone methyltransferase, isolated microspore culture

## Abstract

Bolting is an important agronomic character of the Chinese cabbage, but premature bolting can greatly reduce its commercial value, yield, and quality. Here, early-bolting mutant 1 (*ebm1*) was obtained from a Chinese cabbage doubled haploid (DH) line “FT,” by using an isolated microspore culture and ethyl methanesulfonate (EMS) mutagenesis. The *ebm1* was found to bolt extremely earlier than the wild type “FT.” Genetic analysis indicated that the phenotype of the *ebm1* was controlled by a single recessive nuclear gene. Using a mapping population of 1,502 recessive homozygous F_2_ individuals with the *ebm1* phenotype, the *ebm1* gene was mapped to between the markers SSRhl-53 and SSRhl-61 on chromosome A04 by using SSR markers, and its physical distance was 73.4 kb. Seven genes were predicted in the target region and then cloned and sequenced; the only difference in the sequences of the *ebm1* and “FT” genes was with *Bra032169*. Unlike that in “FT,” the *Bra032169* in *ebm1* had a novel 53 bp insertion that caused the termination of amino acid coding. The mutation was not consistent with EMS mutagenesis, and thus, may have been caused by spontaneous mutations during the microspore culture. Based on the gene annotation information, *Bra032169* was found to encode the histone methyltransferase *CURLY LEAF* (*CLF*) in *Arabidopsis thaliana*. *CLF* regulates the expression of flowering-related genes. Further genotyping revealed that the early-bolting phenotype was fully co-segregated with the insertion mutation, suggesting that *Bra032169* was the most likely candidate gene for *ebm1*. No significant differences were noted in the *Bra032169* expression levels between the *ebm1* and “FT.” However, the expression levels of the flowering-related genes *FLC*, *FT*, *AG*, and *SEP3* were significantly higher in the *ebm1* than in the “FT.” Thus, the mutation of *Bra032169* is responsible for the early-bolting trait in Chinese cabbage. These results provide foundation information to help understand the molecular mechanisms of bolting in the Chinese cabbage.

## Introduction

Bolting is a crucial stage in the transition from vegetative to reproductive growth in *Cruciferous* crops and is regulated by various internal regulatory and external environmental factors (Srikanth and Schmid, 2011; Andrés and Coupland, 2012; Cho et al., 2017). For crops to adapt to their climates, they must bolt at the appropriate time, and premature bolting as a result of environmental conditions and genetic factors, can seriously affect crop yields and quality.

For *Brassica rapa* crops, bolting is more important than flowering, because it can accurately represent the node of transition from vegetative growth to reproductive growth (Nishioka et al., 2005). Several models have been proposed that explain the molecular mechanisms underlying bolting, the main ones being the florigen theory, the nutrient diversion hypothesis, and the multifactorial control model (Yong et al., 2000). In *Arabidopsis thaliana*, studies have shown that a total of six regulatory pathways that are involved in flowering time affect bolting, including the vernalization, photoperiodism, gibberellin, autonomous, aging, and ambient temperature pathways (Fornara et al., 2010; Srikanth and Schmid, 2011; Whittaker and Dean, 2017). These pathways were shown to be mutually connected, forming an intricate regulatory network affecting flowering. Among these, the vernalization and photoperiodism pathways are the two most important regulatory pathways. New molecular regulatory mechanisms underlying flowering time are yet to be revealed (Chen and Penfield, 2018; Qin et al., 2019).

A total of 180 genes related to flowering time were found in *A. thaliana* (Fornara et al., 2010). The regulatory pathways involved in flowering are closely related and influence each other. They were all found to act on specific flowering-related genes to induce the transition of the plant from one growth stage to another. These flowering-related genes were named as floral integrator genes (Simpson and Dean, 2002) and included *FT* (*Flowering Locus T*), *SOC1* (*SUPPESSOR OF OVEREXPRESSION OF CONSTANS 1*), and *LFY* (*LEAFY*) (Zhang Y. N. et al., 2014).

Previous studies on the genetic mechanisms of bolting in Chinese cabbage showed that naturally breeding materials were mostly used as research materials. Bolting is a complicated quantitative trait, controlled by multiple genes, and easily affected by environmental conditions (Elers and Wiebe, 1984; Nishioka et al., 2005; Wang and Zhu, 2011). Nowadays, quantitative trait locus (QTL) analysis or genome wide association study are carried out using segregated and natural populations, and numerous bolting-related QTLs have been identified (Wu et al., 2012; Li et al., 2015; Wang et al., 2018; Xi et al., 2018; Xiao et al., 2019). Three major genes affecting bolting, *BrFLC1*, *BrFLC2*, and *BrVIN3.1*, have previously been cloned and validated (Xiao et al., 2013; Dechaine et al., 2014; Su et al., 2018).

Chinese cabbage (*Brassica rapa* L. ssp. *pekinensis*) is part of the A genome class of the *Brassica* genus and is an important vegetable crop in China and other parts of east Asia. Bolting is an important agronomic trait in *B. rapa* crops. Premature bolting can result in non-heading, reduce the commercial value, and seriously affect the yield and quality of the Chinese cabbage. Therefore, the study of the genetic mechanisms controlling the bolting trait in Chinese cabbage is of great importance.

To explore new ways to elucidate the molecular mechanisms of the bolting trait, we created an early-bolting mutant 1 (*ebm1*) of Chinese cabbage via mutagenesis. The genetic linkage map of *ebm1* was constructed using SSR molecular markers. Fine mapping of the mutated gene in *ebm1* was conducted and a candidate gene was predicted. Thus, our results lay the foundation for elucidating the molecular mechanisms of the bolting trait in Chinese cabbage.

## Materials and Methods

### Plant Materials

The *ebm1* plants was obtained from a doubled haploid (DH) line “FT” of Chinese cabbage by a combination of isolated microspore culture and ethyl methanesulfonate (EMS) mutagenesis; the mutant was diploid, as determined by flow cytometry, and exhibited stable inheritance after multiple generations (Huang et al., 2016a).

### Mutant Trait Evaluation

In the spring of 2017, 30 wild-type “FT” plants (10 plants per replication, with three replications each) and 30 *ebm1* plants (the same as that above) were selected for bolting trait identification under natural conditions. The BI (bolting index), DE (days to 5 cm elongated stalk), DE10 (days to 10 cm elongated stalk), and FT (flowering time) were recorded; the measurements were performed according to the method described by Yu et al. (2004) and Liu et al. (2016). The BI was an identification method for bolting, based on bud appearance, bolting, and flowering, and six grades for the bolting investigation and five grades for the bolting identification were established. The BI was evaluated when the first fully opened flower was visible in the wild-type “FT” and *ebm1* plants, according to the method of Liu et al. (2016).

### Genetic Analysis and Construction of Mapping Population

The *ebm1* and its wild-type “FT” were used as the parents. The segregation of the F_1_, F_2_, and BC_1_ generations were observed and recorded at the bolting stage, based on the DE index in *ebm1* and the wild-type “FT.” Finally, the genetic characteristics of the bolting were analyzed.

The *ebm1* and a Pak-choi (*Brassica rapa* L. ssp. *chinensis*) DH line “13A516,” which exhibited late-bolting, were used as the parents to construct the mapping population. At the bolting stage, the recessive homozygous individuals with the early-bolting phenotype (based on the DE index in *ebm1*) in the F_2_ generation were selected as the mapping population under natural conditions.

### DNA Extraction, PCR Amplification, and Development of Molecular Markers

The young leaves of the parents (*ebm1* and Pak-choi DH line “13A516”) and the mapping population of the F_2_ generation were used for gene mapping, and the genomic DNA was extracted using the cetyltrimethyl ammonium bromide (CTAB) method, as described by Murray and Thompson (1980), with minor modifications.

PCR amplification was performed according to the method of Huang et al. (2016b), and the PCR products were analyzed on a 5% denaturing polyacrylamide gel and silver stained. A total of 176 SSR markers evenly distributed on 10 chromosomes across the *Brassica* A genome, and they were used to screen the polymorphisms between the *ebm1* and “13A516.” After identification of the linkage markers, new SSR markers were further designed using Primer Premier 5.0 software; the sequences of the designed SSR markers are listed in Supplementary Table S1.

### Linkage Analysis and Genetic Map Construction

Linkage analysis of the *ebm1* with molecular markers was performed using JoinMap 4.0 and the Kosambi mapping function.

### Cloning and Sequence Analysis of Candidate Genes

Within the target region, all the genes were identified using the *Brassica* Database^1^. The cloning and sequence analysis of the candidate genes were conducted as described by Huang et al. (2016b). Primers were used to amplify the coding sequence (CDS) and promoter (∼2000 bp) of the candidate gene; and the primers have been listed in Supplementary Tables S2–S4.

### Phylogenetic Analysis

The amino acid sequence of the candidate gene was acquired from the *Brassica* Database, and those of its homologous genes in other species were further obtained from the National Center for Biotechnology Information database^2^. Based on the similarities of the amino acid sequences, the phylogenetic tree was constructed using the MEGA6 software.

### Expression Analysis of the Candidate Genes

Quantitative reverse-transcription PCR (qRT-PCR) was used to identify the expression of the candidate gene in the wild-type “FT” and *ebm1* plants. Total RNA was isolated from the leaves and flowers using the RNApure Total RNA kit (Aidlab, Beijing, China). The first-strand cDNA was synthesized using HiScript IIQ RT SuperMix (Vazyme, Nanjing, China). The *Actin* gene and *18S rRNA* gene were used as the internal reference control (Huang et al., 2015; Chen et al., 2016). The qRT-PCR was performed using the QuantStudio 6 Flex Real-Time PCR System (ABI, United States).

The expression of flowering-related genes, such as *FLC* (*Flowering Locus C*), *FT*, *AGAMOUS* (*AG*), *AGAMOUS LIKE 19* (*AGL19*), and *SEPALLATA 3* (*SEP3*), was examined in the flowers and leaves of the wild-type “FT” and *ebm1*. The relative expression levels of the genes were calculated using the 2^–ΔΔ*Ct*^ method (Livak and Schmittgen, 2001). All reactions were performed using three technical and biological replications. The differences in gene expression were analyzed using OriginPro8.0 software. The primers used for the qRT-PCR analysis are listed in the Supplementary Table S5.

## Results

### Morphological Characterization of *ebm1*

**FIGURE 1:**
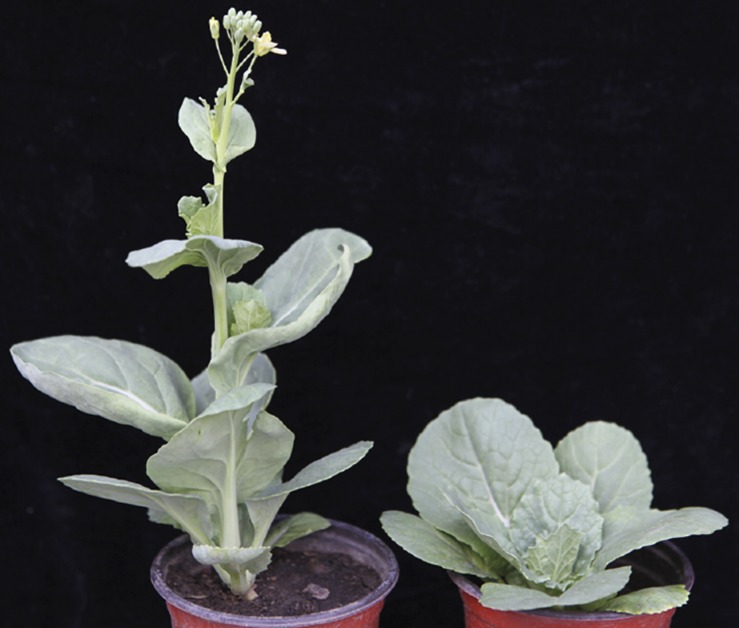
Phenotype characterization of wild-type “FT” (right) and *ebm1* (left) plants.

Compared with the wild-type “FT,” the *ebm1* exhibited the early-bolting phenotype, with slightly curled leaves (Figure 1). The BI indicated that the *ebm1* belonged to the extremely early-bolting type, and the three other indices of bolting, DE, DE10, and FT, differed significantly between the *ebm1* and wild-type “FT” (Table 1).

**TABLE 1:** Determination of the indicators for bolting traits between the wild type “FT” and *ebm1* plants.

Materials	BI	DE	DE10	FT
“FT”	67.49 ± 0.40	63.50 ± 0.26	67.42 ± 0.67	68.38 ± 0.40
*ebm1*	88.13 ± 0.55*	45.76 ± 0.49*	50.60 ± 0.70*	51.47 ± 0.46*

BI, bolting index; DE, days to 5-cm-high elongated floral stalk; DE10, days to 10-cm-high elongated floral stalk; FT, flowering time. Means and standard error (SE)-values were calculated from three independent replicates. Data were analyzed using SPSS16.0 (Chicago, IL, United States). *Significantly different at a level of 0.05 by t-test.

### Genetic Analysis of *ebm1*

The reciprocal cross F_1_ plants exhibited the same phenotype as the wild-type “FT,” indicating that the early-bolting phenotype of the *ebm1* was controlled by nuclear genes. The F_2_ population had late bolting, with an early-bolting ratio of 3.11:1 (χ^2^ = 0.07 < χ^2^0.05 = 3.84), consistent with the Mendelian ratio of 3:1. In addition, the segregation ratio of the F_1_ × *ebm1* in the BC1 progenies was ∼~1:1 (χ^2^ = 0.09 < χ^2^0.05 = 3.84) (Table 2). These results demonstrated that the phenotype of *ebm1* was controlled by a single recessive nuclear gene.

**TABLE 2:** Genetic analysis of the early bolting trait in the Chinese cabbage *ebm1* plants.

Generation	Late bolting	Early bolting	Total	Segregation ratio	Expected ratio	χ^2^
P_1_ (“FT”)	99	0	99			
P_2_ (*ebm1*)	0	98	98			
F_1_ (P_1_ × P_2_)	180	0	180			
F_1_ (P_2_ × P_1_)	182	0	182			
BC_1_ (F_1_ × “FT”)	187	0	187			
BC_1_ (F_1_ × *ebm1*)	85	89	174	0.96: 1	1:1	0.09
F_2_	165	53	218	3.11: 1	3:1	0.07

### Identification of SSR Markers Linked to the *ebm1* Gene

There were 176 SSR markers that were evenly distributed across 10 linkage groups of the *Brassica* A genome, which were used to detect the polymorphisms between the *ebm1* and “13A516.” Of the 176 SSR markers, 81 exhibited polymorphisms between the two parents. Thirty recessive homozygous individuals from the mapping population of the F_2_ generation were selected to determine whether they were linked with the target gene. The marker cnu_m244a, located on chromosome A04, showed linkage with the *ebm1* gene.

**FIGURE 2:**
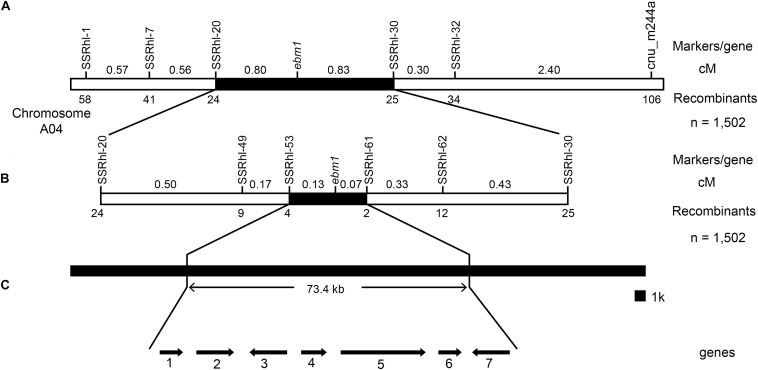
Genetic and physical maps of the *ebm1* gene. **(A)** Preliminary mapping of the *ebm1* gene locus. **(B)** Fine mapping of the *ebm1* gene. **(C)** Candidate genes in the target region. 1–7: *Bra032173–Bra032167*. Arrows indicate the direction of gene expression. The number of recombinants between the markers and *ebm1* is indicated under the linkage map; numbers above the linkage map present the genetic distance between the adjacent markers in centimorgans (cM).

New SSR markers close to the cnu_m244a locus were designed and developed, and the results showed that the polymorphic markers SSRhl-1, SSRhl-7, SSRhl-20, SSRhl-30, and SSRhl-32 were tightly linked to the *ebm1* gene. A total of 1,502 recessive homozygous individuals with the *ebm1* phenotype in the F_2_ generation were investigated as the mapping population. The results indicated that the recombinant individuals selected using markers SSRhl-30 and SSRhl-32 were included in those for cnu_m244a, however, the recombinant individuals selected using markers SSRhl-1, SSRhl-7, and SSRhl-20 were completely different from those for cnu_m244a, suggesting that they were located on the other side of the *ebm1* gene. Based on the recombination frequency statistics, the *ebm1* gene was found to be located between the markers SSRhl-20 and SSRhl-30, at a genetic distance of 0.80 and 0.83 cM, respectively (Figure 2A and Supplementary Figure S1).

### Fine Mapping of the *ebm1* Gene

To further narrow the target region, new SSR markers were designed and developed between the markers SSRhl-20 and SSRhl-30. Of these, markers SSRhl-49, SSRhl-53, SSRhl-61, and SSRhl-62 were tightly linked to the *ebm1* gene, based on the distribution of the recombinant individuals, revealing that the *ebm1* gene was located between the markers SSRhl-53 and SSRhl-61. The marker SSRhl-61 showed two recombinants with the *ebm1* gene, and four recombinants were detected between SSRhl-53 and the *ebm1* gene; the closest markers were SSRhl-61 and SSRhl-53 and they were 0.07 and 0.13 cM away from the *ebm1* gene, respectively. The physical distance between these two markers was estimated to be ~73.4 kb (Figure 2B and Supplementary Figure S1).

### Cloning and Sequencing Analysis of the Candidate Genes in the Target Region

**FIGURE 3:**
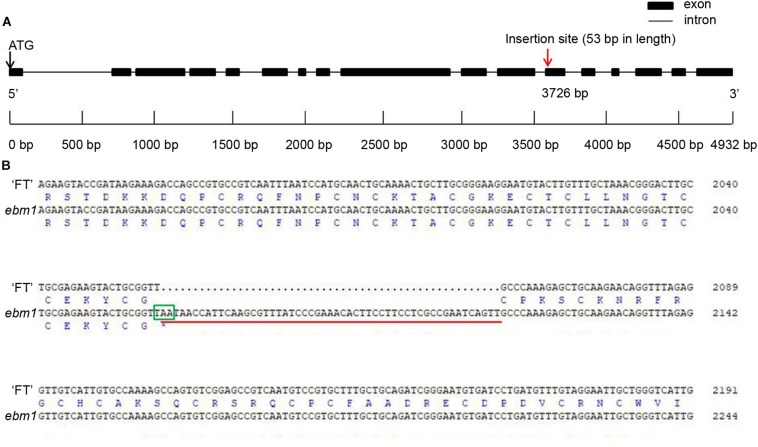
Gene structure and sequence alignment analysis. **(A)** Gene structure of *Bra032169* and the insertion site. **(B)** Alignment of encoding sequences and amino acid sequences of *Bra032169* between wild-type “FT” and *ebm1* plants. Red underline, insertion points of the 53 bp sequences; Green frame, the position where amino acid coding was prematurely terminated.

Seven genes were predicted to be present in the target region using the *Brassica* Database (Figure 2C). These seven genes were cloned and sequenced, and the results showed that the only differences between the *ebm1* and wild-type “FT” existed in *Bra032169*. Unlike that in the wild-type “FT,” a 53 bp insertion occurred in the *ebm1* (Supplementary Figure S2), and the insertion site was located in the 12th exon (A04: 10,754,930) of *Bra032169*. Owing to this insertion, the codon at the insertion site was changed from TGC (Thr) to TTT (U), causing the termination of the amino acid coding in the *ebm1*. The CDS of *Bra032169* is 2777 bp and encodes a protein of 686 amino acid residues in the *ebm1* (Figure 3). The CDSs of the other genes evaluated in the mapping region showed no difference. In addition, the upstream 2.0 kb promoter sequence of *Bra032169* was cloned, and the results showed that the promoter sequence did not differ between the *ebm1* and wild-type “FT.”

### Co-segregation and Candidate Gene Analysis

Annotation information for the seven genes in the target region was obtained from the *Brassica* Database (Supplementary Table S6). *Bra032169* was determined to encode a histone methyltransferase CURLY LEAF (CLF), which is an important H3K27 methyltransferase (Schönrock et al., 2006; Zhang et al., 2007; Jiang et al., 2008; Hennig and Derkacheva, 2009). In *A. thaliana*, CLF (*AT2G23380*) is involved in the control of leaf and flower morphogenesis. Mutations in the *CLF* gene can result in an early-flowering phenotype with curled leaves (Berná et al., 1999; Serrano-Cartagena et al., 2000).

Genotype analysis of 49 recombinants between the two closely linked markers, SSRhl-20 and SSRhl-30, based on the results of primary mapping, indicated that the early-bolting phenotype was co-segregated with the insertion mutation (Supplementary Figure S3). *Bra032169* was, thus, predicted to be the most likely candidate gene responsible for the *ebm1* phenotype.

A phylogenetic tree was constructed to understand the phylogenetic relationship between these species (Supplementary Figure S4). Amino acid sequence identity between the Chinese cabbage CLF and other homologs varied from 98 to 100%. The amino acid sequence alignment showed a high similarity (98%) with the CLF in *A. thaliana*. Proteins that share significant homology with CLF are found in *Brassica* and other species. This sequence similarity among CLF and its homologs suggested that CLF is highly conserved and may serve a conserved function in various organisms.

### Expression Analysis of the Candidate Gene

**FIGURE 4:**
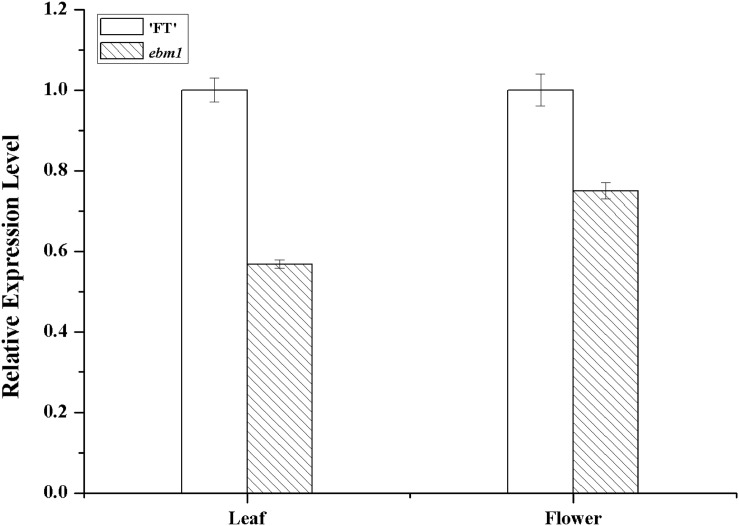
Expression level of *Bra032169* in the leaf and flower of wild-type “FT” and *ebm1* plants. Statistical analysis was performed using *t*-tests with significant differences at the 0.01 level.

To examine the expression patterns of the *Bra032169* gene in *ebm1*, qRT-PCR analysis was performed using the leaf and flower tissues, and no significant difference was noted between the *ebm1* and wild-type “FT” (Figure 4).

Previous studies have shown that CLF can regulate the expression of flowering-related genes, such as *FLC*, *FT*, *AG*, *AGL19*, and *SEP3*, by methylation modification, thus, regulating flowering in *A. thaliana* (Bowman et al., 1989; Goodrich et al., 1997; Schubert et al., 2006; Schönrock et al., 2006; Saleh et al., 2007; Zhang et al., 2007; Jiang et al., 2008; Crevillén et al., 2014).

**FIGURE 5:**
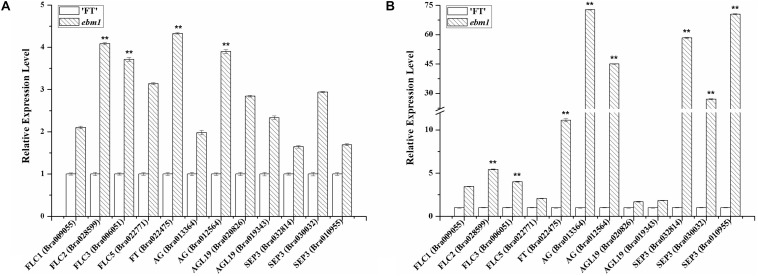
Expression levels of the flowering-related genes in the wild-type “FT” and *ebm1* plants. **(A)** Flower. **(B)** Leaf. **Significantly different at the 0.01 level by *t*-test.

To investigate whether the *ebm1* gene affected the expression of flowering-related genes, we evaluated the expression of *FLC*, *FT*, *AG*, *AGL19*, and *SEP3* in the flowers and leaves of the *ebm1* and wild-type “FT.” The expression of all these flowering-related genes was higher in the *ebm1* than in the wild-type “FT.” The expression of *FLC*, *FT*, *AG*, and *SEP3* was significantly higher, suggesting that these genes may influence the early-bolting phenotype in the *ebm1* (Figure 5).

## Discussion

Bolting is an important agronomic characteristic of Chinese cabbage, but premature bolting can seriously reduce its commercial value, yield, and quality. Therefore, understanding the molecular mechanisms that underlie bolting is important for Chinese cabbage. In the present study, we characterized the *ebm1*, which exhibited early bolting with slightly curled leaves, derived from a Chinese cabbage DH line “FT,” by using an isolated microspore culture combined with EMS mutagenesis. The genetic backgrounds of the *ebm1* and wild-type “FT” were inferred to be highly consistent, and the differences mainly occurred at the mutation sites; thus, the *ebm1* is an ideal material for the study of the molecular mechanisms underlying bolting in Chinese cabbage. Genetic analysis demonstrated that *ebm1* was controlled by a single recessive nuclear gene. Using 1,502 recessive homozygous individuals with the *ebm1* phenotype of the F_2_ generation as the mapping population, the *ebm1* gene was found to be located between the markers SSRhl-61 and SSRhl-53 that were the closest, on chromosome A04. The physical distance was estimated to be 73.4 kb, containing seven predicted genes in the target region. The insertion mutation within the 12th exon of *Bra032169* was co-segregated with the early-bolting trait; in conjunction with the results of the CDSs and the annotation information, *Bra032169* was predicted to be the most likely candidate gene for the *ebm1*. *Bra032169* encodes a histone methyltransferase CLF, the loss of which can result in the early-flowering phenotype with curled leaves in *A. thaliana* (Goodrich et al., 1997). To our knowledge, this is the first study to show that the histone methyltransferase CLF corresponds to the early-bolting trait in *B. rapa*.

Naturally breeding materials have been used to investigate the genetic mechanisms of bolting in the Chinese cabbage. Bolting was considered to be a quantitative trait, and numerous QTLs related to bolting time have been located (Ajisaka et al., 1995; Zhang et al., 2006, 2015; Li et al., 2009; Kakizaki et al., 2011; Zhang Y. N. et al., 2014; Liu et al., 2016; Wang et al., 2018). In the present study, the early-bolting trait of *ebm1* was found to be a quality character and controlled by a single gene, suggesting that the mutant gene of the *ebm1* might be the major gene related to bolting. This is of vital significance for the study of node genes regulating flowering transition in Chinese cabbage.

*Bra032169* was predicted to be the candidate gene for the *ebm1*, and the sequencing results showed that the insertion mutation occurred in the CDS of *Bra032169*, resulting in the termination of the amino acid coding. The common types of gene mutation resulting from EMS mutagenesis mainly include point mutation, base pair deletion, and chromosome fragment deletions (Greene et al., 2003). However, the mutation detected in the present study was clearly not consistent with what is expected after EMS mutagenesis. This may be explained by the fact that the *ebm1* mutation was obtained via isolated microspore cultures, as some studies have shown that during this process, microspores develop to the callus by dedifferentiation, and the regenerated plants are then derived via redifferentiation. At the callus stage, the parenchyma cells are in the indeterminate status, and the callus cells are easily affected by external conditions and can be mutated during redifferentiation. Generally, the external causes of this type of spontaneous variation may be the types and proportions of hormones and carbon sources in the culture medium (Guo et al., 2014; Kadhimi et al., 2014) or the times of subculture (Nehra et al., 1992). These factors can cause gene mutations (Neelakandan and Wang, 2012), chromosomal abnormalities (Zhang D. et al., 2014), and transposon activation (Azman et al., 2014) when acting on the callus cells, thus, generating regenerated plants with mutant phenotypes. The mutation rate caused by gene mutations is relatively high in the spontaneous mutation type of tissue culture, while the injury of regenerated plants caused by gene mutation is relatively slight, facilitating stable inheritance in the offspring. Therefore, we speculated that the insertion mutation of *Bra032169* in *ebm1* may have been caused by a spontaneous mutation during the isolated microspore culture, rather than by the EMS mutagenesis.

Flowering is controlled by various epigenetic factors, which activate or repress the transcription of flowering genes (He, 2012). Previous studies have shown that not only was *CLF* involved in the leaf and flower morphology but that it also played an important role in the regulation of flowering time (Goodrich et al., 1997; Chen et al., 2010). In the present study, the qRT-PCR analysis showed that the expression of *Bra032169* in the leaves and flowers had no significant difference between the *ebm1* and wild-type “FT.” It has been reported that the variation in promoter sequences could affect the gene expression in plants (Wang et al., 2003; Xia et al., 2006; Li et al., 2019). The upstream 2.0 kb promoter sequence of *Bra032169* showed no difference between the *ebm1* and wild-type “FT,” which was consistent with the results of the qRT-PCR analysis. Since the insertion mutation in the *Bra032169* caused the termination of the amino acid coding in the *ebm1*, we speculated that the mutation had no influence on the expression of *Bra032169*, however, loss-of-function proteins may be produced, thus, affecting the regulation of its downstream genes. In *A. thaliana*, loss of *CLF* causes early flowering, a reduction in global H3K27me3 levels, and increased expression of *FLC* and *FT*, as well as several floral homeotic genes, including *AG*, *AGL19*, and *SEP3* (Schönrock et al., 2006; Schubert et al., 2006; Adrian et al., 2010; Lopez-Vernaza et al., 2012; Liu et al., 2018; Wu et al., 2018). Payá-Milans et al. (2019) isolated a tilling mutant line, *braA.clf-1* in yellow sarson (*Brassica rapa* ssp. *trilocularis*), exhibiting the smaller and curled leaves and defective floral organs. ChIP (chromatin immunoprecipitation) and qRT-PCR analysis showed that the floral identity gene *AG* had high expression level due to reduced H3K27me3 in the leaves of *braA.clf-1* mutant. Given this, we further analyzed the expression patterns of these flowering-related genes between the *ebm1* and wild-type “FT.” *FLC*, *FT*, *AG*, and *SEP3* were significantly higher in the *ebm1* than in the wild-type “FT,” which was consistent with the results of previous studies (Schönrock et al., 2006; Schubert et al., 2006; Lopez-Vernaza et al., 2012). The *AG*, *FT*, *SEP3*, and *FLC* genes have been shown to be necessary for the early-flowering phenotype of *clf* mutants (Mizukami and Ma, 1992; Goodrich et al., 1997; Pelaz et al., 2001), and these target genes of *CLF* have antagonistic effects on the flowering time (Lopez-Vernaza et al., 2012). Of these, *FT*, *AG*, and *SEP3* promote flowering, while *FLC* inhibits flowering in the *clf* mutants. Importantly, the effects of the elevated *FLC* expression are masked by increased FT activity in the *clf* mutants, causing the early-flowering phenotype. These results revealed that the phenotype of the *clf* mutants represented a balance between these antagonistic factors (Schönrock et al., 2006; Schubert et al., 2006; Lopez-Vernaza et al., 2012). Therefore, we speculated that the mutation of *Bra032169* may affect the expression of *FLC*, *FT*, *AG*, and *SEP3*, and the antagonistic interactions of these genes influenced flowering time, eventually causing the early-bolting phenotype in the *ebm1*. Additional evidence is needed to elucidate the functions of *CLF* in controlling flowering time in the *ebm1*.

To conclude, the *ebm1* gene was mapped to a 73.4 kb genomic region on the A04 chromosome, and *Bra032169* was predicted to be the candidate gene that is mutated in the *ebm1*. Further exploration of the function of the candidate gene is required to characterize the molecular mechanisms underlying bolting in the Chinese cabbage.

## Data Availability Statement

The datasets generated for this study can be found in the GenBank under accession numbers BankIt2283090 Seq1 MN708212 and BankIt2283090 Seq2 MN708213.

## Author Contributions

HF and SH designed the experiments and supervised this study. SH and LH performed the research and wrote the manuscript. WF, CL, ZL, and XL participated in the research and data analysis. All authors approved the manuscript.

## Conflict of Interest

The authors declare that the research was conducted in the absence of any commercial or financial relationships that could be construed as a potential conflict of interest.

## Footnotes

1 http://brassicadb.org/brad/

2 https://www.ncbi.nlm.nih.gov/

## Supplementary Material

FIGURE S1 Examples of markers tightly linked to *ebm1* (TIF). **(a)** Polymorphisms screened by part of the SSR primers between the two parents. **(b)** Recombinant individuals with SSRhl-30 in the mapping population. **(c)** Recombinant individuals with SSRhl-20 in the mapping population. **(d)** Recombinant individuals with SSRhl-61 in the mapping population. **(e)** Recombinant individuals with SSRhl-53 in the mapping population. M, Marker; P_1_, “13A516”; P_2_, *ebm1*; *, recombinant individuals.

FIGURE S2 Examination of the PCR products of the candidate gene *Bra032169* by agarose gel electrophoresis (TIF). M, Marker D2000.

FIGURE S3 Alignment of the cDNA sequences of *Bra032169* in the 49 F_2_ recombinant individuals, “FT,” and mutant *ebm1* (TIF). Forty-nine early-bolting individuals represent the F_2_ recombinants of the two closely linked markers, SSRhl-20 and SSRhl-30, based on the results of the primary mapping.

FIGURE S4 Phylogenetic tree of the ebm1 protein in the Chinese cabbage and its homologs in other species (TIF).
