# Supplementary figures and images for: An Insertion Mutation in Bra032169 Encoding a Histone Methyltransferase Is Responsible for Early Bolting in Chinese Cabbage (Brassica rapa L. ssp. pekinensis)
Source: Front Plant Sci. 2020 May 12;11:547. doi: 10.3389/fpls.2020.00547 (PMC7235287; doi:10.3389/fpls.2020.00547)

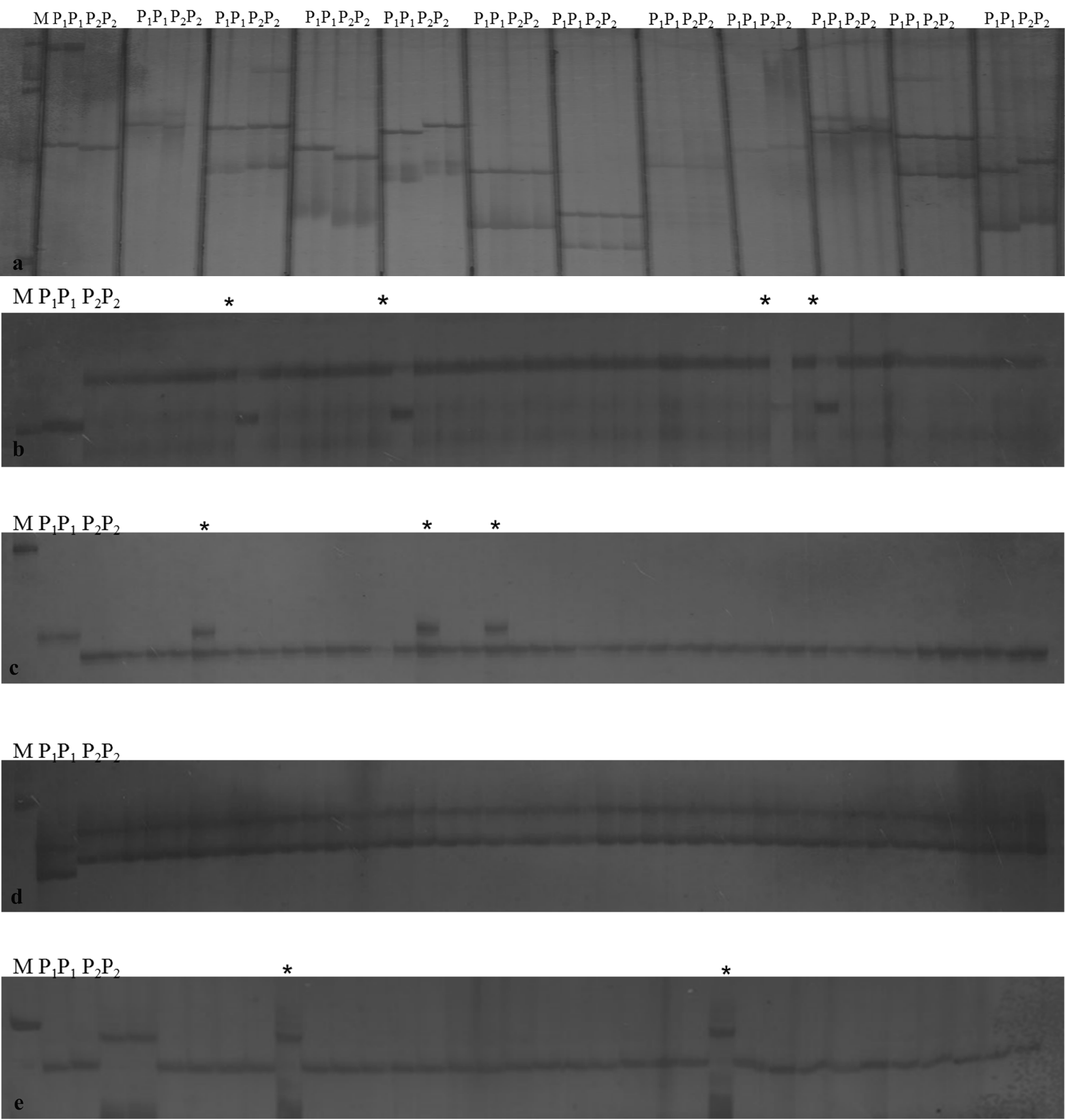

Supplement: FIGURE S1 — Examples of markers tightly linked to ebm1 (TIF). (a) Polymorphisms screened by part of the SSR primers between the two parents. (b) Recombinant individuals with SSRhl-30 in the mapping population. (c) Recombinant individuals with SSRhl-20 in the mapping population. (d) Recombinant individuals with SSRhl-61 in the mapping population. (e) Recombinant individuals with SSRhl-53 in the mapping population. M, Marker; P1, “13A516”; P2, ebm1; *, recombinant individuals. [file Image_1.TIF]

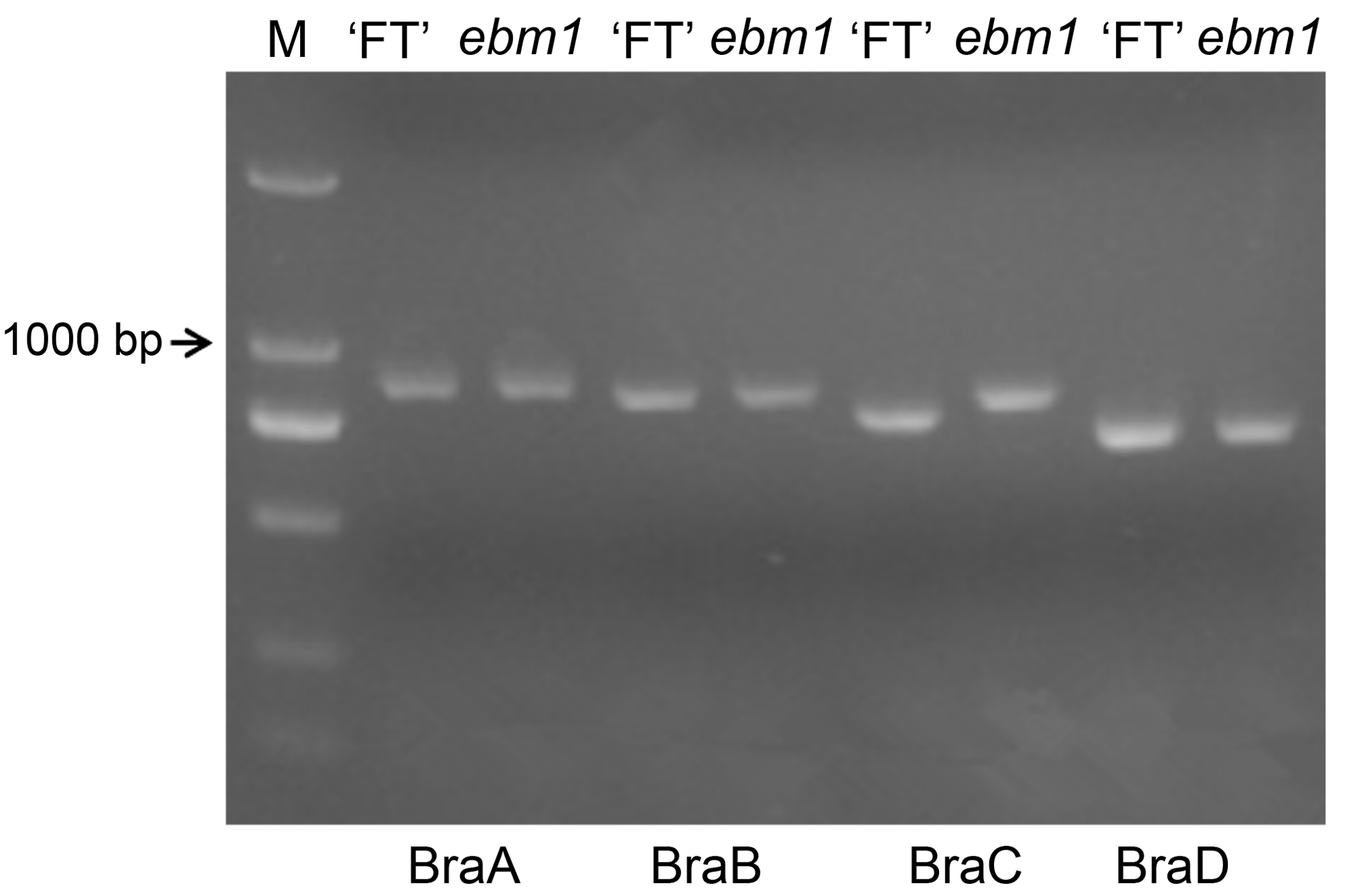

Supplement: FIGURE S2 — Examination of the PCR products of the candidate gene Bra032169 by agarose gel electrophoresis (TIF). M, Marker D2000. [file Image_2.TIF]

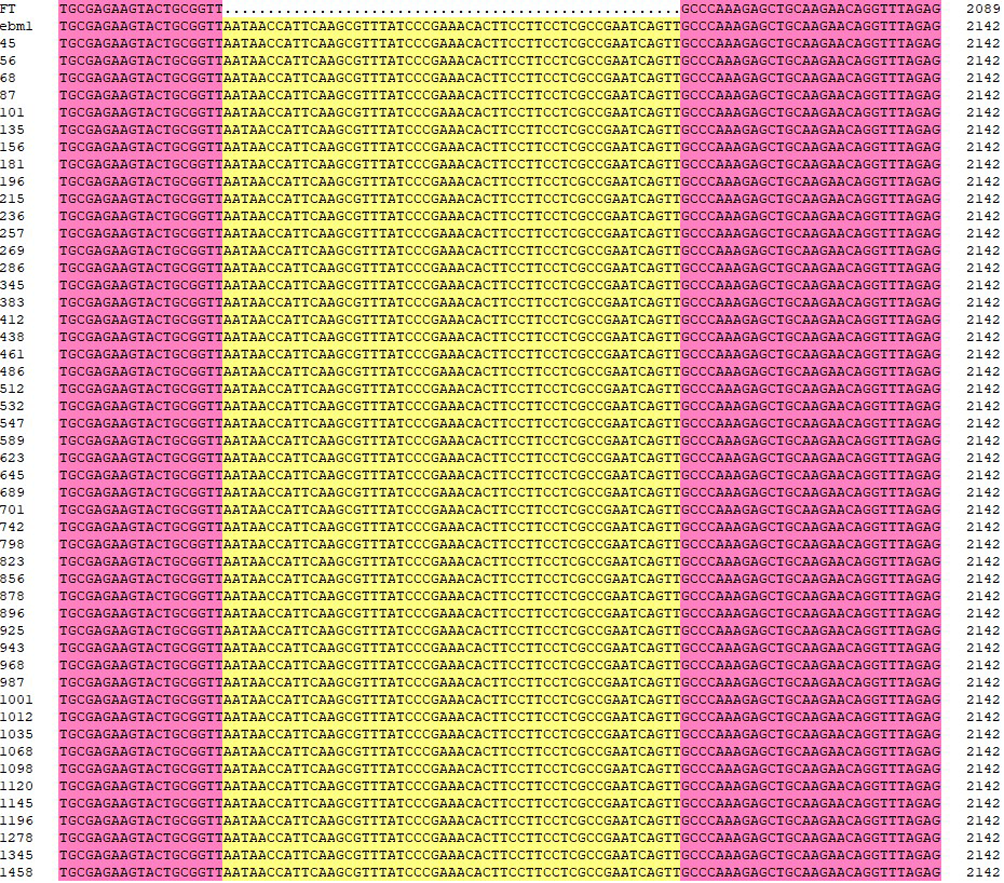

Supplement: FIGURE S3 — Alignment of the cDNA sequences of Bra032169 in the 49 F2 recombinant individuals, “FT,” and mutant ebm1 (TIF). Forty-nine early-bolting individuals represent the F2 recombinants of the two closely linked markers, SSRhl-20 and SSRhl-30, based on the results of the primary mapping. [file Image_3.TIF]

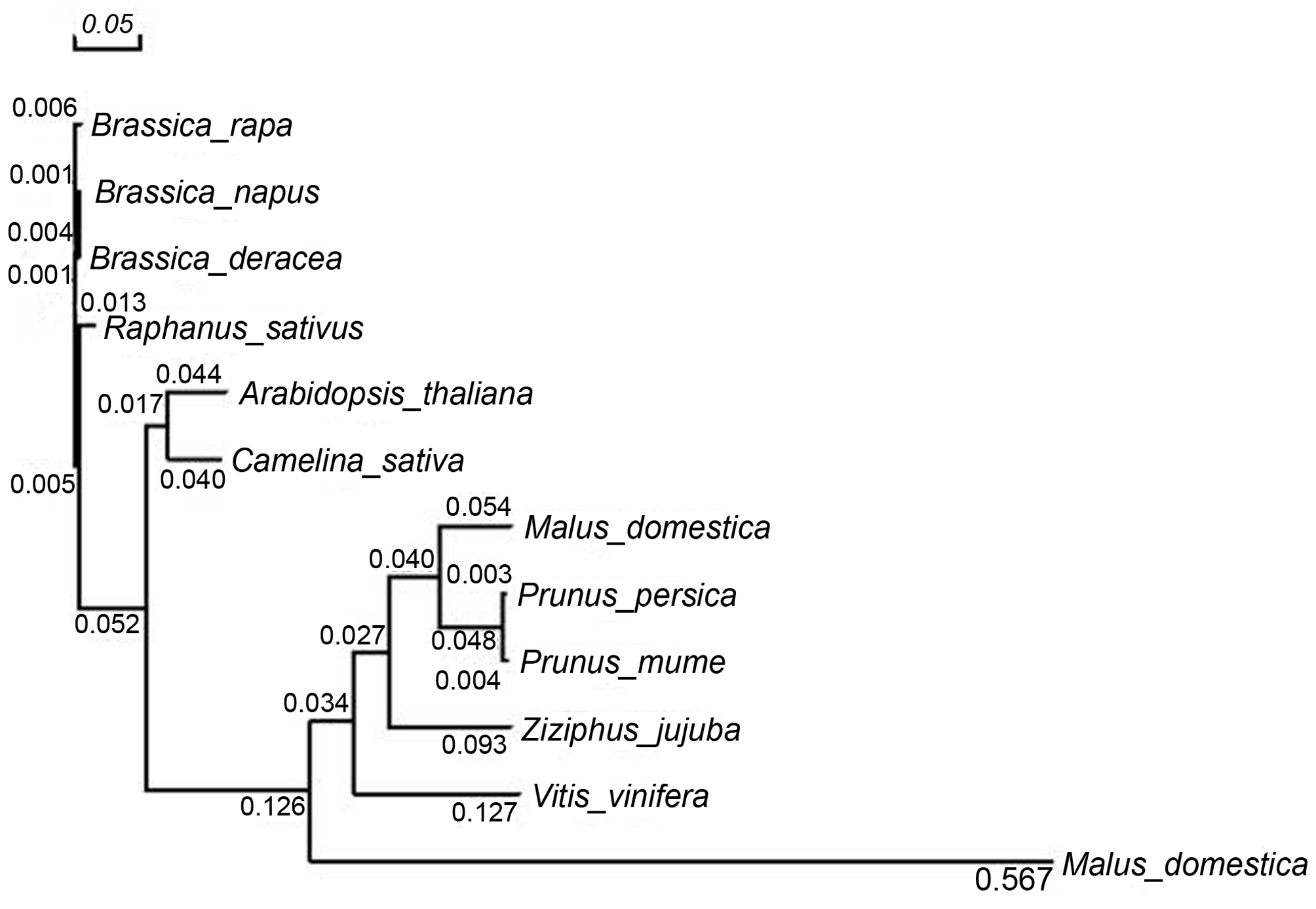

Supplement: FIGURE S4 — Phylogenetic tree of the ebm1 protein in the Chinese cabbage and its homologs in other species (TIF). [file Image_4.TIF]
