# Supplementary material for: An Insertion Mutation in Bra032169 Encoding a Histone Methyltransferase Is Responsible for Early Bolting in Chinese Cabbage (Brassica rapa L. ssp. pekinensis)
Source: Front Plant Sci. 2020 May 12;11:547. doi: 10.3389/fpls.2020.00547 (PMC7235287; doi:10.3389/fpls.2020.00547)
Supplement: Supplementary file 5 [file Table_1.DOCX]

Table S1 Primer sequences of SSR markers

| Marker | Primer Sequences | | Tm (°C) |
| --- | --- | --- | --- |
|  | Forward (5′-3′) | Reverse (5′-3′) |  |
| cnu_m244a | CGGAGATAACCGGAATGGAA | GGATGCTCTGAGACACCCAAA | 57 |
| SSRhl-1 | ATGCGGGTAAAGTGGTGGA | TTCTTCGCCGGGTCGT | 57 |
| SSRhl-7 | TGTCCATAATTAGCTGCAATGC | GAAATAAACTCCAATTCTACTATAGAGTTC | 57 |
| SSRhl-20 | TCTTCACTAGCACAATCGTGC | GACAACATAAGAAACCTCAAAAACA | 57 |
| SSRhl-30 | TGAACAATATCGAAACAGTCCTC | CAACAACCATGCTACAATCTGC | 57 |
| SSRhl-32 | AATAGTGGGTCTTACGAACTTCTAGA | GATGCGATACATGAATCAACGA | 57 |
| SSRhl-49 | ATGCGTTTTCATCTGATTCCA | GGGAAAGGAAAATTGATGGAG | 57 |
| SSRhl-53 | CCCACACACAAAATTTCTTCC | ATGGAACGGAGGGAGTATGTTA | 57 |
| SSRhl-61 | CCTTTTCCTCTTCTCTTCCCTT | TTCATCTTCAATGGCATCTTCA | 57 |
| SSRhl-62 | TGACTGACTCACTAGACTTGTAATCTG | CATCATTCAATTGTTTCAGTGTCT | 57 |
