# Supplementary material for: An Insertion Mutation in Bra032169 Encoding a Histone Methyltransferase Is Responsible for Early Bolting in Chinese Cabbage (Brassica rapa L. ssp. pekinensis)
Source: Front Plant Sci. 2020 May 12;11:547. doi: 10.3389/fpls.2020.00547 (PMC7235287; doi:10.3389/fpls.2020.00547)
Supplement: Supplementary file 6 [file Table_2.DOCX]

Table S2 Primers for the coding sequences of candidate gene *Bra032169*

| Primer name | Primer Sequences | | Length of PCR products (bp) | Tm (°C) |
| --- | --- | --- | --- | --- |
|  | Forward (5′–3′) | Reverse (5′–3′) |  |  |
| Bra032169A | CCGCACAAGCACTCTACGA | GGCAATCAAACACAAGGCA | 841 | 57 |
| Bra032169B | TGGCGATAATCAAGCAGAGA | TACATTTTCACCCACAAACTCTT | 811 | 57 |
| Bra032169C | CTTCTCAGAAGCATGTAAAATCTG | CAAGCGTACCATCCCCAC | 795 | 57 |
| Bra032169D | CATGCAACTGCAAAACTGCT | CTAAGCAACCTTCTTGGGTC | 743 | 57 |
