# Supplementary material for: An Insertion Mutation in Bra032169 Encoding a Histone Methyltransferase Is Responsible for Early Bolting in Chinese Cabbage (Brassica rapa L. ssp. pekinensis)
Source: Front Plant Sci. 2020 May 12;11:547. doi: 10.3389/fpls.2020.00547 (PMC7235287; doi:10.3389/fpls.2020.00547)
Supplement: Supplementary file 7 [file Table_3.DOCX]

Table S3 Primers for the promoter sequences of candidate gene *Bra032169*

| Primer name | Primer Sequences | |
| --- | --- | --- |
|  | Forward (5′–3′) | Reverse (5′–3′) |
| Bra032169-P1 | CTATCTTTG GCACGGTTTATCA | CCATAAGACCCTACCTCTACCG |
| Bra032169-P2 | TACGGTAGAGGTAGGGTCTTATGG | TACGGTAGAGGTAGGGTCTTATGG |
| Bra032169-P3 | GTGTGAGTGATCGCTGAGATGA | GGTGGCGGAAGAAGA AGG |
