# Supplementary material for: An Insertion Mutation in Bra032169 Encoding a Histone Methyltransferase Is Responsible for Early Bolting in Chinese Cabbage (Brassica rapa L. ssp. pekinensis)
Source: Front Plant Sci. 2020 May 12;11:547. doi: 10.3389/fpls.2020.00547 (PMC7235287; doi:10.3389/fpls.2020.00547)
Supplement: Supplementary file 8 [file Table_4.DOCX]

Table S4 Primers for the coding sequences of other genes in the mapping region

| Primer name | Primer sequence | |
| --- | --- | --- |
|  | Forward (5′-3′) | Reverse (5′-3′) |
| Bra032173 | ACCCTTTCGCCGCTCAC | GCGCATCGTAGAGACAAACAT |
| Bra032172A | CACAAAATGGCTCAGGTTCAA | CCCAATCCCACTTCTACGAG |
| Bra032172B | AAGAAGGCACTGAAGAAGCTAAAC | CACGCAAAGCAAACAAGACTG |
| Bra032172C | TCAGTCTTGTTTGCTTTGCGT | GAATCATCTTGTGCCATGTATTTT |
| Bra032172D | GCAGCCACAGGTTAGTCAGTT | GAGAAAACAATCATTCCGCAA |
| Bra032171A | ACAACACTTCTACTTTTCTTCGGA | TTGTCGTGGAAACAGAGGCT |
| Bra032171B | CGAGCCGCTGTGGAGATA | CCATATAAGGATGCTGGTTTTTT |
| Bra032171C | AACTCCATCACTTCCAGACCG | ACCGCTCGTTTCACAGTCTC |
| Bra032171D | GAAGTGGCAGAGACTGTGAAAC | ATCCACCGACCTTGTTGC |
| Bra032170A | GAAACACATGAGCGACGAGAG | AGAAGCTGTCCGAGAATACATACTT |
| Bra032170B | AAGACTCTGAGGAATGCTTTGAAC | TGAAGAAACCGCTGCAATACAC |
| Bra032170C | CGGTTTCTTCAGACCTGTTACG | TGCCTCCATTTCCCATTTTAT |
| Bra032170D | AGGAGAAACCTCAAGCAGTAGATAA | CAACATCTTCTTAGGTGGCAAAT |
| Bra032168 | AACACTCGCTAAGTCCACCGT | GGAATGTCCAATACCAGGGCT |
| Bra032167A | ATCAAGTGGTAAAAAGTGCGTT | AGGATGTTGGTGGACTTGATG |
| Bra032167B | AGGATGTTGGTGGACTTGATGT | ATTACCGAACTGCCGTGG |
| Bra032167C | GCTTTGAACCACGGCAGT | TGAGGCTATTGCTAATTGTAAGG |
| Bra032167D | ACCTCCTTACAATTAGCAATAGCC | GCAGGAAGGTTATCACCGTTA |
