# Supplementary material for: An Insertion Mutation in Bra032169 Encoding a Histone Methyltransferase Is Responsible for Early Bolting in Chinese Cabbage (Brassica rapa L. ssp. pekinensis)
Source: Front Plant Sci. 2020 May 12;11:547. doi: 10.3389/fpls.2020.00547 (PMC7235287; doi:10.3389/fpls.2020.00547)
Supplement: Supplementary file 9 [file Table_5.DOCX]

Table S5 Primers used for qRT-PCR

| Gene | Primer name | Forward sequence (5’-3’) | Reverse sequence (5’-3’) | Annotation for *B.rapa* | Blast to *A.[thaliana](javascript:;)* |
| --- | --- | --- | --- | --- | --- |
| *CURLY LEAF* | CLF | TACCGATAAGAAAGACCAGCC | GCAGCAAAGCACGGACATT | *Bra032169* | *AT2G23380* |
| *FLOWERING LOCUS C 1* | FLC1 | TGGACTGTGGTTCACACCAT | GCGTTCTCAAGGTGTTCCTC | *Bra009055* | *AT5G10140* |
| *FLOWERING LOCUS C 2* | FLC2 | TCGCTCTTCTCGTTGTCTCA | TCATCAGCATGTTGTTTTCCA | *Bra028599* | *AT5G10140* |
| *FLOWERING LOCUS C 3* | FLC3 | CGACAAGTCACCTTCTCCAA | GGAGGAGGAGACAACGAGAA | *Bra006051* | *AT5G10140* |
| *FLOWERING LOCUS C 5* | FLC5 | GTTGGGCTTCTCGTTGTCTC | AGGGCATTGAGATCATCAGC | *Bra022771* | *AT5G10140* |
| *FLOWERING LOCUS T* | FT | AGAGGTGACAAATGGGTTGG | CTCGGAGGTGAGGATTGCTA | *Bra022475* | *AT1G65480* |
| *AGAMOUS* | AG | GCCAAATTGCGTCAACAAAT | CTGCCTTCCAAGTTCCTGAG | *Bra013364* | *AT4G18960* |
| *AGAMOUS* | AG | AACCGTGGCAGAAATTAACG | TGCCTTCCAAATTCCTAAGC | *Bra012564* | *AT4G18960* |
| *AGAMOUS-LIKE 19* | AGL19 | TGTGATGCTGAAGTTGCTTTG | CCTCGCTCCAACTGATTCTC | *Bra020826* | *AT4G22950* |
| *AGAMOUS-LIKE 19* | AGL19 | TCTCAGCAAGCAAGAGACGA | CCTCGCTCCAACTGATTCTC | *Bra019343* | *AT4G22950* |
| *SEPALLATA 3* | SEP3 | TGCAGAGGTTGCTCTCATCA | GCCTCTCTTGAAGGCACATT | *Bra032814* | *AT1G24260* |
| *SEPALLATA 3* | SEP3 | ATGATCCGGACACTGGAGAG | TAAGGCTTCGTAACGCTCCT | *Bra030032* | *AT1G24260* |
| *SEPALLATA 3* | SEP3 | TGCCTTCAAGAGAGGCCTTA | GGTCCAAGATCTTCTCCCAAT | *Bra010955* | *AT1G24260* |
| *ACTIN* | ACTIN | CGAAACAACTTACAACTCCA | CTCTTTGCTCATACGGTCA |  |  |
| *18S rRNA* | 18SrRNA | GTTCTTAGTTGGTGGAGCGATTT | ACCTGTTATTGCCTCAAACTTCC | |  |
