# Supplementary material for: An Insertion Mutation in Bra032169 Encoding a Histone Methyltransferase Is Responsible for Early Bolting in Chinese Cabbage (Brassica rapa L. ssp. pekinensis)
Source: Front Plant Sci. 2020 May 12;11:547. doi: 10.3389/fpls.2020.00547 (PMC7235287; doi:10.3389/fpls.2020.00547)
Supplement: Supplementary file 10 [file Table_6.DOCX]

Table S6 Candidate genes within the mapping region

| Gene ID | Start | End | Gene Annotations (BLASTX to *Arabidopsis thaliana*) | E value |
| --- | --- | --- | --- | --- |
| *Bra032173* | 10,726,580 | 10,727,098 | AP2 domain-containing transcription factor | 3.00E-65 |
| *Bra032172* | 10,732,730 | 10,735,519 | PAB4, PABP4; PAB4 (POLY(A) BINDING PROTEIN 4); RNA binding / translation initiation factor | 0.00E+00 |
| *Bra032171* | 10,736,932 | 10,739,406 | protein kinase family protein | 0.00E+00 |
| *Bra032170* | 10,746,796 | 10,749,069 | transport protein-related | 0.00E+00 |
| *Bra032169* | 10,751,315 | 10,756,246 | CLF, ICU1, SDG1, SET1; CLF (CURLY LEAF); transcription factor | 0.00E+00 |
| *Bra032168* | 10,771,356 | 10,771,607 | unknown protein | 5.00E-24 |
| *Bra032167* | 10,790,982 | 10,793,699 | protein kinase family protein | 0.00E+00 |
